# Modulating parallel photon avalanche in Ho^3+^ for multicolor nanoscopy and related applications

**DOI:** 10.1038/s41377-025-02033-3

**Published:** 2025-10-02

**Authors:** Dingxin Huang, Yung Doug Suh, Guanying Chen

**Affiliations:** 1 MIIT Key Laboratory of Critical Materials Technology for New Energy Conversion and Storage, School of Chemistry and Chemical Engineering, Harbin Institute of Technology, Harbin, 150001 China; 2 Key Laboratory of Micro-systems and Micro-structures, Ministry of Education, Harbin Institute of Technology, Harbin, 150001 China; 3 Department of Chemistry, Ulsan National Institute of Science and Technology (UNIST), Ulsan, 44919 South Korea; 4 Center for Multidimensional Carbon Materials (CMCM), Institute for Basic Science (IBS), Ulsan, 44919 South Korea

**Keywords:** Imaging and sensing, Multiphoton microscopy, Nanoparticles, Super-resolution microscopy, Nonlinear optics

## Abstract

Tuning the emissive chromaticity of parallel photon avalanches in Ho^3+^-doped nanoparticles with dual reservoir levels enables multicolor super-resolution imaging under 965 nm single wavelength continuous-wave excitation.

Photon avalanche is a special kind of luminous phenomenon featured by ultra-high optical nonlinearity ($N\ge 15$) at the excitation threshold, where emission surges with a tiny increase of excitation power. This luminous mechanism significantly advances the development of various biophotonic applications, including high-sensitivity detection and bioassay, efficient microlaser, and sub-diffraction nanoscopy^1–6^. In particular, using fluorescent probes capable of generating photon avalanche emission can directly achieve a leap in imaging spatial resolution, scaling as $\sqrt{N}$, beyond the diffraction limit even on a conventional confocal microscope. Though photon avalanche for super-resolution imaging has been realized in several trivalent lanthanide ion (Ln^3+^) nano-systems at room temperature^5,6^, taking advantage of long-lived energy levels in Ln^3+^, the lack of tunability for emission chromaticity greatly restricts the practical deployment of photon avalanche in multicolor nanoscopy and the relevant research on behaviors of multiple biological targets. Thus, the photon avalanche scheme, that there are individual emission channels without spectral crosstalk under single wavelength laser beam excitation, is worthy of further exploration among Ln^3+^ ions.

Now, writing in *Nature Photonics*, Dong et al.^7^ report tunable emission spectra profile of photon avalanche in Ho^3+^-doped nanoparticles for multicolor sub-diffraction nanoscopy. The emissive tunability is attributed to parallel photon avalanche (PPA) based on two reservoir levels, ^5^I_7_ and ^5^I_6_, with long lifetime in the order of milliseconds, of holmium ion (Ho^3+^). By co-doping with Ce^3+^/Tm^3+^ to adjust the population of energy levels in two photon avalanche loops, nanoparticles of distinct chromaticity were engineered to label subcellular fractions—imaging spatial resolution of 102 nm and 78 nm has been achieved in Red and Green/Blue channels, respectively, at different excitation power densities of 965 nm single wavelength continuous-wave (CW) laser.

The lateral spatial resolution of microscopy is theoretically determined by the wavelength of the excitation source ($\lambda$), the numerical aperture of the objective lens (${NA}$), and optical nonlinearity ($N$), with the relationship of $\lambda /(2* {NA}* \sqrt{N})$^8^. Therefore, a high-resolution optical microscope is always equipped with an oil-immersed objective lens of high numerical aperture. Although a shorter excitation wavelength can enhance imaging resolution, optimal performance requires balancing resolution gains with sufficient tissue penetration depth; hence, using a light source in the first biological window (650–1000 nm) is a wise choice for in vivo microscopic imaging.

Among numerous fluorescent probes, lanthanide-doped materials with ladder-like metastable energy levels can generate upconversion luminescence efficiently under excitation of a near-infrared (NIR) CW laser diode, which facilitates deep-tissue imaging without background noise interference^9^. In all upconversion mechanisms, photon avalanche exhibits the highest optical nonlinearity that primarily depends on the relative magnitude of the excited-state absorption (ESA) rate compared to the ground-state absorption (GSA) rate. For high-order nonlinearity, the absorption cross-section of the ESA process must be 10^4^ times higher than that of the GSA process. In the specific implementation scheme, the excitation wavelength needs to be adjusted according to the energy level structure of the lanthanide ions used to meet the above requirements.

Besides, the cross relaxation (CR) to accelerate the population of the intermediate energy level is a key process in photon avalanche. Specifically, one lanthanide ion at a high-energy level can bring about two ions at a long-lived intermediate energy level through donating partial energy to a nearby ion in the ground state. Combined with an efficient ESA process, this positive-feedback loop would dramatically amplify the population of high-energy levels and the corresponding upconversion emission. However, it should be noted that excessive CR will cause a loss of the population of high-energy level, so the doping concentration of lanthanide ions, related to the rate of CR, should be optimized to achieve a higher-order nonlinearity.

For nanoscale material, its relatively large ratio of surface area to volume, especially when the diameter of nanoparticle goes down 20 nm^10^, leads to high defect density in a single nanoparticle, which depopulates the emission energy level and lowers the optical nonlinearity. To inhibit the quenching induced by this surface defect, the universal solution is coating inert shell layers with a thickness above 3 nm^11^. Integrating all the train of thought, Dong et al. designed core/shell nanoparticles NaGdF_4_:10% Ho@NaYF_4_ (13.5@3.1 nm), which realize multicolor photon avalanche with ~20th order optical nonlinearity at sub-25 kW/cm^2^ irradiance of 965 nm CW laser. The prolonged rise time (~350 ms) at excitation threshold reflects the slow build-up of reservoir populations prior to avalanche triggering. With excellent photo-stability of 4f–4f transition for lanthanide ions, such a photon avalanche without overlap in emission spectra is suitable for long-term multiplex sub-diffraction microscopy.

The breakthrough performance that large optical nonlinearity with a mild excitation threshold stems from a dual-reservoir energy-looping mechanism in holmium-doped nanoparticles, as shown in Fig. 1, where intermediate metastable ^5^I_7_ and ^5^I_6_ energy levels of Ho^3+^ ions act as parallel reservoirs to drive photon avalanche of different emissive levels synchronously via CR pathways amplified by high doping concentration (10% Ho^3+^). By contrast, conventional photon avalanche is sustained by only one reservoir level and correspondingly one primary emissive level is activated, which poses inherent obstacles to modulation of photon avalanche emission chromaticity and multicolor super-resolution imaging application^5,6^.

**Fig. 1:**
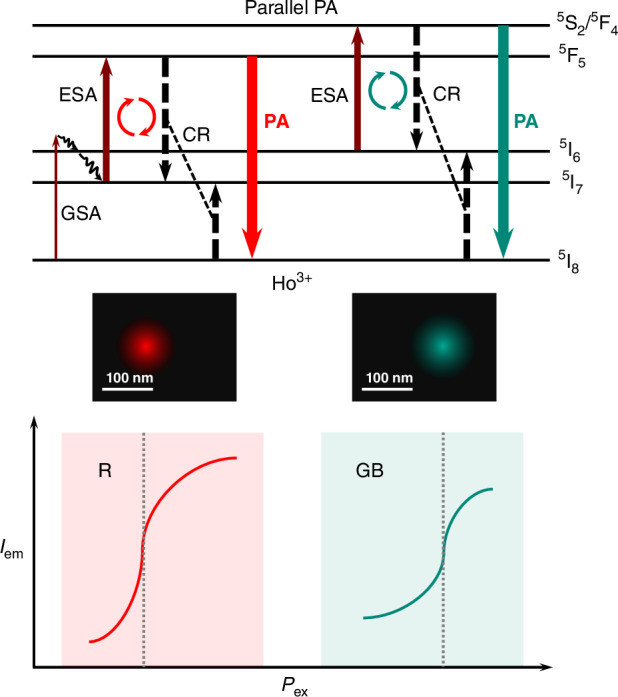
Parallel photon avalanche in Ho3+ enables multicolor nanoscopy under 965 nm continuous-wave excitation. Top: Schematic diagram of the parallel photon avalanche (PA) mechanism. Rare population on dual reservoir levels (^5^I_6_ and ^5^I_7_), originated from phonon-assisted GSA (multiphonon relaxation process is indicated by curved lines with an arrow), would increase exponentially at their excitation thresholds through the energy-looping process, which is composed of excited-state absorption (ESA) from reservoir level to emissive level (^5^F_5_ and ^5^S_2_/^5^F_4_) as well as cross relaxation (CR) between emissive level and ground state (^5^I_8_). The energy-looping process involved in green–blue (red) PA is indicated by clockwise circles with cyan (red) color. GSA, ground-state absorption. Solid line with an arrow, absorption or emission; dashed lines with an arrow linked by a thinner dashed line, cross relaxation. Middle–Bottom: Schematic sketch of multicolor sub-diffraction imaging enabled by PA emissions in red (R) and green–blue (GB) channels at different excitation power density (*P*_ex_). *I*_em_, emission intensity. For each imaging channel, the optimal imaging resolution of the designed nanoparticle is achieved at the *P*_ex_, indicated by dotted lines, where the function of *I*_em_ to *P*_ex_ has the greatest slope. Diffraction limit is about 333 nm (full width at half maximum) when applying an objective lens with a numerical aperture of 1.45. White scale bar, 100 nm. Note that all the images are artificially drawn ones to help readers’ understanding

Utilizing PPA nanoparticles doped with Ho^3+^ as the fluorescent probe, a common confocal/multiphoton laser-scanning microscope could be promptly and directly upgraded to a multicolor imaging platform with sub-100 nm spatial resolution, which also has the potential to detect tiny changes in the physiological environment based on the great sensitivity of PPA. By integrating single low-phototoxicity NIR excitation (965 nm), multicolor capability, and sub-diffraction resolution into a single-nanoparticle platform, PPA technology transcends the limitations of conventional super-resolution techniques (e.g., STED relying on another high-power depletion laser at a specific wavelength)^4,12^, establishing a universal strategy for high-fidelity nanoscale multiplexing in biological systems.

Despite Dong and colleagues have successfully demonstrated a new paradigm of PPA nanoparticles for multicolor nanoscopy^7^, there are some aspects that need to be further explored to promote the application of photon avalanche in biophotonics. First, developing the PPA nanoparticles under shorter wavelength excitation in the first biological window (650–1000 nm) to improve imaging resolution and reduce the absorption of excitation energy by water, which would not only lower the thermal damage caused by the excitation light but also enhance the imaging depth. Second, coupling sensitizer materials with a large absorption cross-section, such as organic dyes or quantum dots, to bring down the excitation threshold of lanthanide-doped PAA nanoparticles, which would also lower the thermal damage caused by the excitation light to a large extent, and provide an approach to adjust the excitation wavelength. But there may be challenges in maintaining stability in long-term irradiation and a complex physiological environment for sensitizer materials. Third, arranging metal nanostructure reasonably around the nanoparticles to shorten the rise time of photon avalanche in favor of fast or large field-of-view imaging, and to enhance the capability of absorbing excitation energy for lanthanide-doped PAA nanoparticles. Fourth, exploiting advanced surface passivation techniques to reduce the size of the nanoparticles while holding the optical nonlinearity for better biocompatibility.

We believe that this landmark work reported by Dong et al.^7^ will inspire the construction of multicolor nonlinear nanomaterials for versatile biophotonic applications, related applications, and beyond.
